# Examining U.S. Newspapers’ Effects on COVID-19 Infection Rates Among Racial/Ethnic Minorities

**DOI:** 10.1089/heq.2021.0142

**Published:** 2022-02-02

**Authors:** Zhan Xu

**Affiliations:** School of Communication, Northern Arizona University, Flagstaff, Arizona, USA.

**Keywords:** health disparities, racial/ethnic disparities, COVID-19, newspapers

## Abstract

**Background:** The coronavirus disease 2019 (COVID-19) pandemic reveals health disparities in the United States. News media are expected to play a major role in reducing racial/ethnic disparities.

**Methods:** Guided by agenda-setting theory in the context of health promotion and the structural approach of media effects, this study assessed the impacts of COVID-19 newspaper articles about racial/ethnic minorities on minorities' infection rates in the early stages of the pandemic, while controlling for social determinants of health (SDOHs).

**Results:** Racial/ethnic minorities are underrepresented in COVID-19 newspaper articles, although newspapers' attention to racial/ethnic minorities' health increased over time. Public exposure to newspaper articles about racial/ethnic minorities was the only significant factor that predicted infection rates among general racial/ethnic minorities. The more the general public in the United States was exposed to related newspaper articles, the lower the infection rates among general racial/ethnic minorities would be. The impacts of SDOHs varied across different racial/ethnic minority groups. Blue states were more likely to be exposed to COVID-19 newspaper articles about racial/ethnic minorities than red states.

**Discussion:** Findings suggest that news exposure to any racial/ethnic group can benefit all minorities. Findings also demonstrate the influence of media agenda on public agenda and policy agenda regarding minority health.

## Introduction

The coronavirus disease 2019 (COVID-19) pandemic in the United States reveals health disparities, especially racial and ethnic disparities. Data indicate that communities of color suffer disproportionately.^1^ As of writing, Blacks are 1.1 times more likely to be infected by COVID-19 and 1.9 times more likely to die from it than non-Latinx Whites. Latinx have 1.3 times higher infection rates and 2.3 times fatality rates compared with non-Latinx Whites.^2^

Health disparities refer to the “differences in health outcomes between groups and their causes among groups of people.” These differences are mainly caused by social determinants of health (SDOHs)—external influences such as social, economic, political, or environmental factors.^3^ Although Healthy People 2020 and 2030 identify eliminating health disparities as a primary goal in the United States,^4^ the progress has remained slow.^5^ The COVID-19 crisis creates a chance to examine and address the factors underlying health disparities.^6^

News media had not effectively communicated the issue of health disparities in the past. Systematic content analysis indicates that racial/ethnic disparities stories only consist of 0.1% to 13.6% of health news.^7^ For instance, 1% of health news from 2000 to 2004 mentioned racial disparities.^8^ From 2005 to 2006, 13.6% of diabetes stories mentioned racial disparities.^9^ During the COVID-19 pandemic, news media, such as print and online newspapers, are expected to play a major role in reducing racial/ethnic disparities. Although several large newspapers (i.e., the *New York Times* and the *Los Angeles Times*) have highlighted this issue on their front page,^1^ the impacts of news stories about racial/ethnic disparities are unclear.

Previous studies demonstrate that media coverage of health disparities can influence health policy, practice, and outcomes.^10,11^ However, few studies have quantified the effects of news articles about racial/ethnic health disparities. This study examined news coverage of racial/ethnic groups in the early stages of the pandemic. It estimated the impacts of related news coverage on racial/ethnic minorities’ health outcomes, while controlling for SDOHs.

### Health disparities and agenda setting in the context of health promotion

Most U.S. adults regard individual health behaviors as the most determining factor for good health.^12^ Moreover, health policy and research mostly focus on health beliefs and behaviors as health outcomes.^13^ This approach ignores SDOHs and emphasizes “individual-oriented causal attributions,” attaching health problems to unhealthy and risky behaviors.^10^ In the context of COVID-19, those unaware of SDOHs may believe that community members refusing to practice social distancing leads to a higher infection rate among their community. However, people of low socioeconomic status may have to live in overcrowded places and may not be able to work remotely, which limits the possibility of social distancing and increases the chance of infection.^14^

Among all the determinants, race/ethnicity, education, and poverty are among the primary determinants^15^ and directly lead to health disparities.^16^ Similarly, data from the U.S. and other countries indicate that older people, people of low socioeconomic status, and racial/ethnic minorities, are more likely to die of COVID-19.^17^ One factor resulting in the limited attention to SDOHs is that the news media rarely discuss them.^18^ According to the agenda-setting theory, frequent exposure to a health issue in news media will increase the awareness and the perceived salience of the issue.^10^

Agenda-setting theory was first introduced to explain the overall news media effects.^19^ Later research indicates a need to develop agenda setting in the context of health promotion. This approach highlights the media's influence on policy and public agendas by actively discussing health issues.^20^ When the media frequently report health disparities, the audience would perceive it as an urgent issue that needs to be addressed. Previous studies demonstrate that news media can increase awareness^21^ and the perceived salience of health disparities.^10^

Regarding the COVID-19 pandemic, a nationally representative survey reveals that those who are exposed to national news, such as *The New York Times*, and *The Washington Post*, are more likely to recognize racial and ethnic health disparities related to COVID-19.^22^ News media also have great impacts on policy agendas. For instance, media can increase policy support to eliminate health disparities.^9^ When media messages frequently frame the issue of health disparities as a high-level public concern, policy makers will be more motivated to address it.^11^

Recent studies reveal that besides increasing the awareness and perceived importance of an issue, news media can also improve health outcomes at the community or societal level. For instance, by examining the news media coverage and annual vaccine rates from 1999 to 2001, a study discovered that news discussing flu shot shortages increased earlier vaccination.^23^

Based on prior research, we may predict that racial/ethnic minorities’ health will be perceived as a severe issue if people are frequently exposed to related news. Minorities may adopt more preventive behaviors after reading related news, leading to better health outcomes. Moreover, exposure to minority health-related news may lead others to advocate for policy change and may also result in more resources to support racial/ethnic minorities. These efforts may also contribute to better health outcomes among minorities.

### Structural approach of media effects and polarizing opinions

According to the structural approach of media effects, media content is a product of a complex interaction among various organizations, groups, individuals working in the media, and audiences. Thus, media content reflects existing structural inequality and may not recognize or emphasize health disparities.^24^ Based on audiences’ interests and needs, some news outlets may prefer the topic of health disparities while others may regard such topics as less newsworthy.^25^ For example, Republicans tend to attribute social status to personal responsibility while Democratic are more likely to acknowledge structural or systemic factors.^26^ As a result, Republicans are less likely to consider racial/ethnic inequality as a critical issue than Democratic.^27^

Therefore, if a news media mainly target Republican audiences who perceive racial/ethnic disparity as less important, the media may not discuss it frequently. In the end, Republican audiences may not be exposed to related news content and may continue to ignore health disparities. Based on the rationale outlined above, we may predict that blue states are exposed to more health news about racial/ethnic minorities than red states and thus are more likely to recognize health disparities, perceive it as more important, have more policy support to eliminate disparities, and finally improve minority health. The impacts of racial/ethnic health disparities’ news on health outcomes are intervened by a state's partisan lean.

In sum, this study aims to examine if frequent exposure to COVID-19 news articles about racial/ethnic minority health will lead to better health outcomes (lower infection rates) among minorities controlling for SDOHs (i.e., race/ethnicity, education, poverty, and state's partisan lean). Besides, this study also investigates whether red states and blue states are exposed to different amounts of COVID-19 news stories about racial/ethnic minorities.

## Methods

### Data collection

Monthly infection rates among Blacks, Latinx, Asians, and Non-Hispanic White in each state were collected from The COVID Tracking Project.^28^ The project started collecting race/ethnicity data from each state in mid-April, 2020. However, not many states reported their data in April. Thus, we collected states’ infection rate data by race/ethnicity in May, June, and July 2020.

To demonstrate the impacts of news articles and exclude the possibility that news articles are influenced by infection rates in the same month, we collected COVID-19 news articles published in April, May, and June 2020, 1 month earlier than the monthly infection rate data.

We first identified all articles with headlines that contained “COVID,” “coronavirus,” “corona,” and/or “SARS” published in national and local U.S. print and online newspapers included in LexisNexis. Then, the daily circulation data of newspapers included in our sample were obtained from Alliance for Audited Media (AAM). The sample included 17,356 news articles published in 63 newspapers (Appendix A1). Finally, among all the COVID-19 news articles published in these newspapers, articles mentioning any specific racial/ethnic group terms (e.g., Latino) or general group terms (e.g., Minority) were obtained (Appendix A2). These terms were adapted from prior health disparity research.^29,30^

### Measures

#### Exposure rate to COVID-19 news

Since news articles can reach and impact readers in different states, a state's infection rate may be affected by COVID-19 news articles published in other states. To more precisely assess media effects, we first calculated a state/district's exposure to COVID-19 news based on the number of COVID-19 news articles published in each newspaper and each newspaper's median daily circulation in the state.

Since state population varies, a state with more exposure to COVID-19 news does not necessarily result in its residents being exposed to more related articles. Thus, exposure rate is critical to examine media effects. We then calculate exposure rate to COVID-19 news based on a state/district's exposure to COVID-19 news and a state's population.^31^ Appendix A3 shows the equations.

#### Exposure rate to minority news

This refers to exposure rate to COVID-19 news about a racial/ethnic minority group. We first calculated a state/district's exposure to minority news based on the number of COVID-19 news articles about a racial/ethnic minority group published in each newspaper and each newspaper's median daily circulation in the state. We then calculate exposure rate to minority news based on a state/district's exposure to COVID-19 news articles about a racial/ethnic minority group and a state's population (Appendix A3).

### SDOHs

SDOHs included population by race/ethnicity (percentage of a minority group among the general population), education (percentage of a minority group with at least a college degree), poverty (percentage of a minority group with income in the past 12 months below poverty level), and age (percentage of a minority group at or over 65). These data were collected from the U.S. Census Bureau.^32^

State partisan lean was another controlled covariate. Each state was categorized into strong Democratic, lean Democratic, competitive, lean Republican, or strong Republican based on the percentage of Democratics and lean Democratics minusing the percentage of Republicans and lean Republicans. A state was strong Democratic if the number was 10% or larger. A state leaned Democratic when the number fell between 5% and 10%. A state was competitive when the difference was between −4% and 4%. A state leaned Republican when the number was within −5% and −10%. A state was strong Republican if the difference was −10% or smaller.^33^ The data were collected from the joint COVID states project.^34^

### Analyses

All analyses are performed using R (version 4.0.2). First, a longitudinal multilevel model was used to examine the association between exposure rate in month *t* and the infection rate among each racial/ethnic minority group in month *t*+1 controlling for sociodemographic covariates. The time lag reveals the impacts of news articles published in month *t* on infection rates in month *t*+1 and excludes the possibility that news articles are influenced by infection rates in the same month. State and month variables were regarded as random effects.

The backward elimination method was used for model selection to “eliminate nonsignificant predictors, reduce the risk of confounding variables in the final model, and improve predictive accuracy.”^35^ In each step, predictors with the largest *p*-value were removed until all predictors that remained in the model were significant (*p*<0.05). In each step when each predictor was removed, *R*^2^ was checked to avoid significant loss of model fit. Finally, the Kruskal–Wallis test was utilized to examine if state partisan lean was associated with the state's exposure rate to COVID-19 news about minorities. Benjamini–Hochberg method was applied to adjust for multiple comparisons in *post hoc* tests.

## Results

A growing number of states reported COVID-19 infection rates among racial/ethnic minorities from May 2020 to July 2020 (Table 1). Specifically, about 80% of states in May, 86% of states in June, and 90% of states in July reported infection rates among at least a minority group. Except for the infection rates among Asians, the infection rates among general minorities, Blacks, and Latinx were higher than the general population. Overall, newspapers published fewer articles related to coronavirus from April 2020 to June 2020 (Table 2). Up to 88% of national and local newspapers mentioned minorities when they reported the pandemic. Less than 22% of the articles mentioned minorities, although an increasing percentage of articles discussed minorities from April to June.

**Table 1.** Infection Rates Among Minorities and the Number of States that Released the Data

	May		June		July	
	No. of states	Median infection rates (range)	No. of states	Median infection rates (range)	No. of states	Median infection rates (range)
General population	44	0.17 (0.004–0.67)	51	0.16 (0.02–0.74)	51	0.32 (0.03–1.44)
General minorites	32	0.33 (0.02–1.49)	38	0.30 (0.01–1)	40	0.41 (0.05–1.32)
Blacks	41	0.28 (0.004–1.41)	43	0.21 (0–1.87)	45	0.43 (0.01–1.19)
Asians	36	0.12 (0–1.58)	41	0.10 (0.01–1.04)	43	0.13 (0.01–0.87)
Latinx	37	0.40 (0.03–1.93)	44	0.42 (0.02–1.63)	46	0.55 (0.05–2.02)

**Table 2.** Number of Newspapers and Coronavirus Disease 2019 News Articles Mentioning Racial/Ethnic Minorities

	April				May				June			
	No. of newspapers	% newspapers	No. of articles	% articles	No. of newspapers	% newspapers	No. of articles	% articles	No. of newspapers	% newspapers	No. of articles	% articles
COVID-19 news articles	60	100	7716	100	60	100	5853	100	63	100	3787	100
Number of articles mentioning racial/ethnic groups	53	88	2312	16	49	82	2388	21	50	79	1645	22
Number of articles mentioning Blacks	48	80	1075	7	46	77	1340	12	47	75	993	14
Number of articles mentioning Asians	29	48	360	2	23	38	333	3	23	37	233	3
Number of articles mentioning Latinx	34	57	263	2	34	57	403	4	31	49	344	5

COVID-19, coronavirus disease 2019.

Regarding the factors that predicted infection rates among general racial/ethnic minorities, the random effects model [F (1, 108)=13.40, *p*<0.001, *R*^2^=0.12] revealed that exposure to COVID-19 news about minorities negatively predicted infection rates among general minorities. Regarding infection rates among Blacks, the model [F (3, 124)=21.03, *p*<0.001, *R*^2^=0.34] revealed that exposure to COVID-19 news about Blacks was not a significant predictor. Percentage of Blacks in the general population and the percentage of people below poverty level among Blacks positively predicted their infection rates.

The percentage of people at or over 65 among Blacks negatively predicted infection rates among Blacks. Regarding infection rates among Asians, the model [F (1, 118)=14.22, *p*<0.001, *R*^2^=0.11] revealed that percentage of people at or over 65 among Asians was a negative predictor. Regarding infection rates among Latinx, the model [F (2, 123)=14.11, *p*<0.001, *R*^2^=0.19] revealed that exposure to COVID-19 news about Latinx was not a significant predictor. Percentage of people at or over 65 among Latinx community and percentage of college degree among Latinx negatively predicted infection rates among Latinx. Detailed information regarding models and whether the proposed arguments were supported appears in Table 3.

**Table 3.** Final Models that Predicted Infection Rates Among Each Racial/Ethnic Minority Group

	General racial/ethnic minorities		Blacks		Asians		Latinx	
Variables	*ß*	*p*	*ß*	*p*	*ß*	*p*	*ß*	*p*
Exposure rate to minority news	−0.1	<0.001	—	—	—	—	—	—
Race/ethnicity	—	—	1	<0.001	—	—		
Education	—	—	—	—	—	—	−9.73	0.05
Poverty	—	—	2.44	<0.001	—	—		
Age	—	—	−5.34	<0.001	−2.13	<0.001	−11.49	<0.001
Proposed argument	Exposure to COVID-19 news articles about general racial/ethnic minorities will lead to lower infection rates among general minorities controlling for SDOHs		Exposure to COVID-19 news articles about Blacks will lead to lower infection rates among Blacks controlling for SDOHs		Exposure to COVID-19 news articles about Asians will lead to lower infection rates among Asians controlling for SDOHs		Exposure to COVID-19 news articles about Latinx will lead to lower infection rates among Latinx controlling for SDOHs	
Whether the proposed argument was supported	Yes		No^a^		No^a^		No^a^	

In both the full models and the final models, exposure rate to minority news refers to exposure rate to COVID-19 news about a racial/ethnic minority group, race/ethnicity refers to the percentage of the minority group among the general population, education means the percentage of the minority group with at least a college degree, poverty refers to the percentage of the minority group with income in the past 12 months below poverty level, and age means the percentage of the minority group at or over 65.

For the general and each racial/ethnic minority group, the full model is: Infection rate among a racial/ethnic minority group=Exposure rate to COVID-19 news+Exposure rate to minority news+Race/ethnicity+Education+Poverty+Age+State Partisan Lean.

^a^ Exposure rate to COVID-19 news articles about Blacks/Asians/Latinx was not significant and therefore was removed during the model building process.

SDOHs, social determinants of health.

Results also demonstrated that state partisan lean was associated with the state's exposure rate to COVID-19 news about minorities (*χ*^2^=31.27, df=4, *p*<0.001). Specifically, strong Democratic (median=2233, range: 357–21,192) and lean Democratic states (median=1861, range: 507–4,067) were more likely than strong Republican states (median=340, range: 82.7–1036) to be exposed to COVID-19 news about minorities (*p*<0.001, *p*=0.002, respectively). Strong Democratic and lean Democratic states were more likely than lean Republican states (median=418, range: 91.9–753) to be exposed to related news (*p*=0.002, *p*=0.02, respectively). Strong Democratic states were more likely than competitive states (median=910, range: 362–2612) to be exposed to related news.

**FIG. 1.**
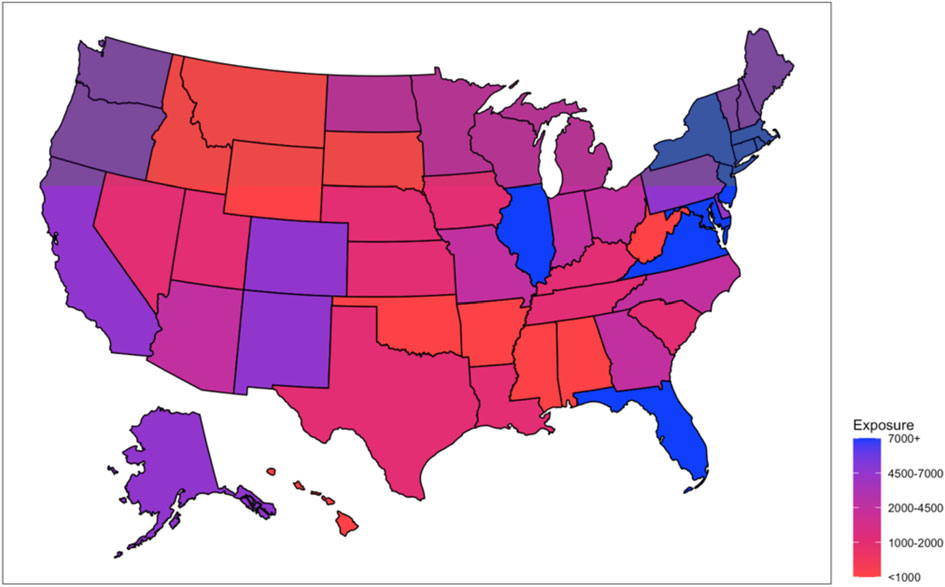
Median exposure rates to COVID-19 news about minorities by state. COVID-19, coronavirus disease 2019.

Table 4 presents the top five and bottom five states based on their median exposure rate to COVID-19 news about minorities. Figure 1 visualizes the exposure rate by state in the United States map.

**Table 4.** States with the Highest and Lowest Median Exposure Rate to Coronavirus Disease 2019 News About Racial/Ethnic Minorities

Rank	State	Median
1	District of Columbia	21,191.61
2	Connecticut	8749.47
3	New York	7989.86
4	Maryland	6750.24
5	New Jersey	5833.61
47	Wyoming	257.80
48	Arkansas	159.51
49	South Dakota	113.65
50	Montana	91.94
51	Mississippi	82.71

## Discussion

Overall, U.S. newspapers decreased their attention to COVID-19 probably because their audiences experienced “news fatigue.”^36^ However, the percentage of articles discussing general racial/ethnic minorities, Blacks, Asians, and Latinx increased, which suggested that newspapers’ attention to minority health increased. At the same time, more states released COVID-19 infection rates among minorities. These findings were consistent with agenda setting in the context of health promotion, implying the association between media agenda and policy agenda. Moreover, these results indicate that minority health is an increasingly critical agenda for the media and policymakers.

Consistent with prior research, when discussing a particular racial/ethnic minority group, the media tend to focus on Blacks.^30^ For instance, this study shows that in June 2020, 14% of COVID-19 news articles mentioned Blacks, whereas only 3% discussed Asians and 5% discussed Latinx. Since Blacks, Asians, and Latinx represent 13.4%, 5.9%, and 18.5% of the U.S. population,^37^ this finding demonstrates that Asians and Latinx are still underrepresented in COVID-19 news. Except for June, Blacks were underrepresented in COVID-19 news published in April and May.

However, results revealed that exposure to each racial/ethnic minority group did not lower the infection rate among the particular group and different amounts of news coverage regarding each minority group did not influence the overall media effects. Exposure to COVID-19 news about general minorities would lower infection rates among general minorities in the following month.

These findings suggest that news exposure to any racial/ethnic group can benefit all minorities. Specifically, with a one unit increase in the exposure rate to COVID-19 news stories about general minorities, the infection rates among general minorities will decrease by 0.1%. For instance, with a total of 23,722,461 minority population in California, if the exposure rate increases 10%, about 2260 minorities would not get infected.

The finding demonstrates the influence of media agenda on public agenda and policy agenda. When news media publish more articles about minority health and more audiences are exposed to the content, more attention will be devoted to health disparities, which may lead to more resources supporting minorities as well as policy and behavior change. These together may reduce infection rates among minorities. Yet, we should note that COVID-19 news stories mentioning minorities may also emphasize personal choice and individual behavior instead of systemic factors. However, this study indicates that only by mentioning minorities, news media can reduce infection rates among general minorities.

This study reveals several SDOHs that had impacts on racial/ethnic minority groups. For example, percentage of people at or over 65 among Blacks, Asians, and Latinx negatively predicted infection rates among these communities. At the early stage of the pandemic, newspapers and studies have highlighted the disproportionate impact on older minorities based on past crises and called for actions that address health equity,^38^ which may contribute to the great attention devoting to the older minority population.

Consistent with previous findings, poorer Blacks are at higher risk for infection mostly due to limited access to safe housing and quality health services.^39^ Moreover, less educated Latinx are more likely to be infected partly because they may have low health literacy, which leads to a lack of health knowledge and less use of preventive measures.^40^ In addition, those with less education, especially Hispanic women, are severely affected by COVID-19 job losses,^41^ which may prevent them from accessing quality health services.

Altogether, these results imply that key determinants of each minority group's health conditions may be different, which highlights the value of tailored messages in health communication. Moreover, policymakers and practitioners should apply a more diverse strategy to address social determinants that affect different racial/ethnic minority groups the most.

Findings must be interpreted in light of several limitations. First, as this study only focused on the effects of print and online newspapers, results may not be generalized to other media channels, such as TV and social media. Next, our sample was limited to newspapers included in LexisNexis and only contained news articles published from April 2020 to June 2020. Thus, the results may not fully reflect the entire scope of COVID-19 news articles that discuss racial/ethnic minorities. Finally, we only examined news articles’ effects in 1 month. Future studies are recommended to explore the long-term effects.

## Abbreviations Used

AAM: Alliance for Audited Media
COVID-19: coronavirus disease 2019
SDOHs: social determinants of health

## Appendix

**Appendix A1.** Newspapers Included in this Study

Anchorage Daily News
Arizona Capitol Times
Bangor Daily News
Central Penn Business Journal
Crain's Chicago Business
Crain's Cleveland Business
Crain's Detroit Business
Crain's New York Business
Daily Herald
Daily Journal of Commerce
Daily News
Daily Record
Dayton Daily News
Deseret News
FDAnews Device Daily Bulletin
FDAnews Drug Daily Bulletin
Finance and Commerce
Idaho Business Review
Lehigh Valley Business
LNP
Long Island Business News
Los Angeles Times
New Orleans CityBusiness
New York Observer
Orange County Register
Pittsburgh Post-Gazette
Providence Journal
Public Opinion
Richmond Times Dispatch
South Bend Tribune
Spokesman Review
St. Louis Post-Dispatch
Star Tribune
Tampa Bay Times
Telegraph Herald
The Atlanta Journal-Constitution
The Baltimore Sun
The Bismarck Tribune
The Columbian
The Daily News of Los Angeles
The Daily Oklahoman
The Decatur Daily
The Evening Sun
The Hartford Courant
The Hill
The Lebanon Daily News
The Mecklenburg Times
The Morning Call
The New York Post
The New York Times
The Pantagraph
The Philadelphia Daily News
The Philadelphia Inquirer
The Salt Lake Tribune
The San Diego Union-Tribune
The Santa Fe New Mexican
The Washington Post
The White House Bulletin
The Wyoming Tribune-Eagle
Tribune-Review
Wall Street Journal Abstracts
Wisconsin State Journal
Yellow Sheet Report

**Appendix A2.** Racial/Ethnic Minority Terms

Native
Indigenous
Asian
Black
African
Hispanic
Latino
Latinx
Ethnicity
Ethnic
Minority
Ethnocentrism
Racism
Race
Racial

### Appendix A3. Equations to Calculate Exposure Rate to Coronavirus Disease 2019 News and Exposure Rate to Minority News

#### Exposure Rate to Coronavirus Disease 2019 News

First, a state/district's exposure to coronavirus disease 2019 (COVID-19) news was calculated using the following equation:

$$E_{i}=\sum_{1}^{k}x_{k}c_{k,i}$$

where *E_i_* is the exposure to COVID-19 news in state *i*. *x_k_* is the number of COVID-19 news articles published in newspaper *k*. *c_k,i_* is *k'*s median daily circulation in state*i*.^A1^

Then, exposure rate to COVID-19 news was calculated using the following equation:

$$R_{i}=E_{i}∕P_{i}\times 1000$$

where *R_i_* is exposure rate to COVID-19 news in state *i*. *E_i_* is exposure to COVID-19 news in state *i*. *P_i_* is most recent population in state*i*.^A1^ State population data were obtained from the U.S. Census Bureau.^A2^

#### Exposure Rate to Minority News

First, a state/district's exposure to minority news was calculated using the following equation:

$$E_{i}=\sum_{1}^{k}x_{k}c_{k,i}$$

where *E_i_* is the exposure to COVID-19 news articles about a racial/ethnic minority group in state *i*. *x_k_* is the number of COVID-19 news articles about a racial/ethnic minority group published in newspaper *k*. *c_k,i_* is *k'*s median daily circulation in state *i*.^A1^ For example, to calculate a state/district's exposure to COVID-19 news about Asians, *E_i_* is the exposure to COVID-19 news articles about Asians in state *i*. *x_k_* is the number of COVID-19 news articles about Asians published in newspaper *k*. *c_k,i_* is *k'*s median daily circulation in state*i*.^A1^

Then, exposure rate to minority news was calculated using the following equation:

$$R_{i}=E_{i}∕P_{i}\times 1000$$

where *R_i_* is exposure rate to COVID-19 news articles about a racial/ethnic minority group in state *i*. *E_i_* is exposure to COVID-19 news articles about a racial/ethnic minority group in state *i*. *P_i_* is most recent population in state*i*.^A1^
